# Dietary Magnesium and Genetic Interactions in Diabetes and Related Risk Factors: A Brief Overview of Current Knowledge

**DOI:** 10.3390/nu5124990

**Published:** 2013-12-06

**Authors:** Adela Hruby, Nicola M. McKeown, Yiqing Song, Luc Djoussé

**Affiliations:** 1 Department of Nutrition, Harvard School of Public Health, 677 Huntington Avenue, Building 2, Boston, MA 02115, USA; E-Mail: ahruby@hsph.harvard.edu; 2 Nutritional Epidemiology Program, Jean Mayer USDA Human Nutrition Research Center on Aging, Tufts University, 711 Washington Street, 9th Floor, Boston, MA 02111, USA; E-Mail: nicola.mckeown@tufts.edu; 3 Department of Epidemiology, Richard M. Fairbanks School of Public Health, Indiana University, 714 N. Senate Avenue, Indianapolis, IN 46202, USA; E-Mail: yiqsong@iu.edu; 4 Division of Aging, Department of Medicine, Brigham and Women’s Hospital, Harvard Medical School, 1620 Tremont Street, 3rd Floor, Boston, MA 02120, USA; 5 Boston Veterans Affairs Healthcare System, Boston, MA 02130, USA

**Keywords:** magnesium, diet, genetic interaction, genome-wide interaction study, diabetes, glucose, insulin

## Abstract

Nutritional genomics has exploded in the last decade, yielding insights—both nutrigenomic and nutrigenetic—into the physiology of dietary interactions and our genes. Among these are insights into the regulation of magnesium transport and homeostasis and mechanisms underlying magnesium’s role in insulin and glucose handling. Recent observational evidence has attempted to examine some promising research avenues on interaction between genetics and dietary magnesium in relation to diabetes and diabetes risk factors. This brief review summarizes the recent evidence on dietary magnesium’s role in diabetes and related traits in the presence of underlying genetic risk, and discusses future potential research directions.

## 1. Introduction

The insights of the last decade into the genetics of type 2 diabetes and impaired glucose and insulin metabolism have yielded dozens of replicated risk loci. The genome-wide association (GWA) era has uncovered additional and novel genetic associations with disease risk that were beyond the purview of the previous genetic era’s linkage or candidate gene studies. However, researchers are now more than aware that GWA and prior approaches have their limitations and, moreover, that our current understanding of the contributions of genetics to complex and multifactorial diseases such as type 2 diabetes must steadily move into ever greater complexity to include gene-gene/gene-environment interactions, epigenetics, whole exome/genome approaches, and beyond.

Among the moves into complexity are investigations in gene-diet interactions, in which diet is considered a form of environmental exposure. A now classic example of a gene-diet interaction study was the reanalysis of the Diabetes Prevention Program data, which assessed the effect of a healthy diet *versus* metformin on diabetes risk in those at risk, in the context of a well-known single nucleotide polymorphism (SNP) at *TCF7L2* (rs7903146) [1]. The results of this re-analysis clearly demonstrated elevated genetic risk at this locus with the TT genotype, but additionally demonstrated that lifestyle changes offset the genetic risk almost entirely. Hundreds, perhaps thousands, of so-called candidate SNP-diet interaction studies have been undertaken, and studies of micronutrient-gene interactions are no exception. Among these micronutrients is, of course, magnesium.

Observational studies and clinical trials have shown that dietary magnesium has relatively consistent beneficial associations with type 2 diabetes and related traits [2,3] or sequelae, from insulin resistance and metabolic syndrome [4], to cardiovascular disease [5]. Coupled to the decade’s findings on the genetics of diabetes are novel findings related to the genetics of magnesium transport and homeostasis, which have provided even more fertile ground for investigating interactions between magnesium and risk of developing diabetes and effects on related phenotypes.

Recently, a handful of investigations have delved into possible genetic interactions between magnesium intake and loci that may be responsible for impaired magnesium metabolism and homeostasis, or impaired glucose and insulin metabolism. These studies have turned up promising and not-so promising research avenues regarding interactions between genetics and dietary magnesium in relation to diabetes and diabetes risk factors.

In this brief review, we summarize the underlying mechanisms thought to drive magnesium’s role in insulin and glucose homeostasis and metabolism, the epidemiologic evidence to date on dietary magnesium in relation to diabetes and related traits, and some recent evidence on magnesium’s role in diabetes in the presence of underlying genetic risk. This review ends with a discussion of research needs and future directions in the magnesium field.

## 2. Basics of Magnesium Homeostasis

Magnesium homeostasis is tightly regulated and serum levels are roughly constant across a wide range of magnesium intake [6]. Serum levels, perhaps because of their clinical measurement universality, dictate diagnoses of hypo- and hypermagnesaemia. However, dietary magnesium correlates with serum magnesium only weakly [7,8]. Age-, sex-, and energy-adjusted correlations of 0.27 including supplement users, and just 0.15 excluding supplement users, have been reported [7], while others have reported no linear association (*r* = 0.05) between self-reported dietary intake of magnesium and levels in serum [8]. Depletion studies [9,10] and supplementation trials [11,12] show that serum magnesium concentrations change slowly, over periods of up to four months from depletion before they stabilize or before the onset of adverse events such as heart arrhythmias or impaired reflexes [10]. Since serum magnesium is not particularly sensitive to intake, except in cases of prolonged deficiency or acute or prolonged hyperalimentation (often drug—e.g., milk of magnesia—or supplement-induced) it is considered a poor marker of dietary magnesium intake. Serum concentrations may therefore not accurately reflect total body magnesium stores, and by the time magnesium deficiency is clinically recognized based on serum concentrations (usually <0.75 mmol/L [6]), an individual’s deficiency may already be moderate to severe [13]. As such, some experts have emphasized the problem of chronic latent magnesium deficiency, which may contribute to the incidence of or exacerbate conditions such as type 2 diabetes and related metabolic disorders, as well as cardiovascular disease and osteoporosis [13,14].

The kidney is the main site of magnesium regulation; magnesium excretion decreases rapidly in response to decreased intake, long before blood concentrations levels fall below the normal range [15]. It follows that any disruption of normal function of the kidneys, or in the presence of renal disease, magnesium homeostasis is also generally impaired. Individuals at specific risk for magnesium deficiency include those with inadequate diets or nutritional supplementation, gastrointestinal disorders with malabsorption, endocrine and metabolic disorders (*i.e.*, type 2 diabetes, hyperparathyroidism, hypoparathyroidism), primary aldosteronism, hungry bone syndrome, as well as those with conditions accompanied by diarrhea or excessive urinary magnesium losses or other renal dysfunction [13,15]. Certain medications, notably proton-pump inhibitors, have also been reported to induce hypomagnesaemia [16].

## 3. Magnesium and Its Putative Mechanisms in Glucose and Insulin Metabolism

Impaired glucose and insulin metabolism lie along the etiologic trajectory that results in type 2 diabetes [17]. While the exact mechanism of magnesium’s role in these processes remains to be elucidated, experimental evidence points to a role for magnesium in both beta-cell dysfunction and in insulin resistance in peripheral tissues. The evidence and hypotheses are briefly summarized below, but readers are referred to the excellent reviews by Günther [18], Bo and Pisu [19], Volpe [4], and Barbagallo and Dominguez [20].

One mechanism through which magnesium may be acting within peripheral tissue is via its effect on tyrosine kinase, a component of the beta subunit of the insulin receptor for which magnesium is a co-factor. Activation of tyrosine kinase produces a signaling cascade that ultimately translocates GLUT4 (the major insulin-regulated glucose transporter expressed in muscle and other insulin-responsive tissues) to the membrane, and allows the cell to take up glucose. Suarez *et al.* [21] reported that alongside a 50% reduction in the insulin sensitivity in rats fed a magnesium-deficient diet, there was also 50% reduced autophosphorylation of the beta subunit of the insulin receptor in isolated gastrocnemius muscle tissue of these rats as compared to controls, and that the tyrosine kinase activity of insulin receptors in these hypomagnesaemic animals was also significantly reduced. In another study, rat epidydmal adipocytes exposed to regular or reduced ambient and intracellular free magnesium ion concentrations showed significantly reduced insulin-stimulated (but not basal) glucose oxidation to carbon dioxide when cultured in low *versus* physiologic magnesium. The authors concluded that their study provides evidence for magnesium’s role distal to glucose entry into the cell, and further, that impaired glucose oxidation may be reversible [22].

Insulin itself may be a regulatory hormone of magnesium metabolism. The mechanism whereby insulin modifies intracellular magnesium is via the activity of ion transport channels, such as Na/H antiporters, calcium-adenosine triphosphatases (Ca-ATPases), and ATPase-dependent pumps. Interestingly, insulin-mediated cellular uptake of magnesium may additionally depend on the activation of the tyrosine kinase of the insulin receptor, as there is evidence that inhibiting the receptor via monoclonal antibody nullifies insulin’s intracellular magnesium-raising effects [20] (notably, low intracellular magnesium does not affect insulin-insulin receptor binding, additionally pointing to a downstream (*i.e.*, tyrosine kinase activation) regulatory focal point [18]). In addition, prolonged high concentrations of circulating insulin, such as those known to occur in insulin resistance, induce increases in renal magnesium excretion, thus perpetuating a deleterious cycle [18]. Barbagallo and Dominguez [20] have examined the relationship between basal intracellular magnesium levels and responsiveness of cells to both insulin and glucose: with lower basal intracellular magnesium concentrations, cells become less responsive to insulin and glucose. Further, they noted that while higher glucose induces magnesium efflux, the lower the basal concentration of magnesium, the less that concentration is responsive to the modifying effects of insulin and glucose—that is, there is a non-linear response and it appears the cells must retain some basal level of magnesium at which they are not as responsive to fluctuations in insulin or glucose. The authors postulate that hyperglycemia induces cellular hypomagnesaemia, which subsequently contributes to the inability of the cell to respond to insulin [20]. It should be noted that the effects of glucose and insulin on intracellular magnesium may not be universal, that is tissue-specific investigations in, for example, heart muscle or erythrocytes, may not be observed in other cells, such as pancreatic islets.

While the exact mechanisms of magnesium’s direct and indirect effects on insulin production and secretion are unknown, several pathways are hypothesized. First, magnesium’s direct role as a cofactor for ATPases affects many steps of the glycolytic pathway. Second, magnesium may be acting as an inhibitor of the inositol 1,4,5-triphosphate (IP3)-gated calcium channel—thus magnesium may be acting as a calcium antagonist [23]. Relatedly, it has been suggested that the calcium-magnesium ratio within cells may be a more powerful regulator of insulin secretion, and that inhibitors and/or potentiators of ion balance or channel activity ultimately regulate insulin secretion [24,25]. A third proposed pathway of magnesium’s actions is via its role in the activation of acetyl-coA carboxylase, which ultimately catalyzes the formation of long-chain fatty acids, which have a role in insulin secretion. For example, in rat islet cells, acetyl-coA carboxylase activity is associated with magnesium in a dose-dependent manner [26]. A fourth mechanism may be related to genomic regulation of transcription. In obese Zucker fatty rats (spontaneous type 2 diabetes animals) fed a magnesium-supplemented diet for 6 weeks beginning at 6 weeks old showed lower fasting and fed-state blood glucose concentrations, better glucose disposal, higher insulin and *C*-peptide concentrations, and increased pancreatic GLUT2 and insulin mRNA expression than animals on the control diet [27]. By 12 weeks of age, all of the eight control animals had developed diabetes while diabetes was present in only one of eight of the magnesium-supplemented animals [27].

Glucose itself likely plays a regulatory role in the magnesium concentration of beta cells. In rat islets, d-glucose (and certain other sugars metabolized by islets) induces a dose-dependent increase in magnesium, independent of insulin release [28]. After experimentation with various inhibitors, researchers concluded that the magnesium-increasing effect of glucose in islets is not merely a consequence of the depolarization of the β-cell membrane (which accounted for approximately one third of magnesium uptake), but also of the islets themselves metabolizing glucose [28].

These mechanisms related to insulin secretion and insulin resistance, coupled with plausible roles of pro-inflammatory cytokines with which low magnesium has also been implicated, may be processes that depend on magnesium both directly and indirectly [18]. Of final mechanistic note, magnesium has long been hypothesized [29] to have a second messenger role with substantial downstream effects. Li *et al.* [30] recently demonstrated that T cells as well as epithelial tissue require free magnesium ion flux for effective antigen receptor signaling, with variable effects on calcium ion flux, depending on the agonist-receptor combination and cell type. Given the complexity and diversity of these signaling mechanisms across different tissues, the specific second messenger role for magnesium in the context of glucose and insulin metabolism remains an interesting and active area of research.

## 4. Epidemiological Evidence for Magnesium in Offsetting Risk of Type 2 Diabetes

Many observational and clinical studies have shown strong associations between serum and dietary magnesium and fasting insulin or insulin resistance [31,32,33,34,35,36,37,38,39,40,41,42,43], and impaired glucose metabolism or type 2 diabetes [2,41,42,43,44,45,46,47,48]. The prospective observational studies of dietary magnesium have been nicely summarized in three separate meta-analyses [3,44,49], all published within the past 6 years. These, alongside the prospective observational literature in type 2 diabetes, are briefly summarized in Table 1, while Table 2 summarizes the observational (primarily cross-sectional) literature on related diabetes traits.

**Table 1:** Prospective observational studies of magnesium intake and risk of type 2 diabetes.

Author (Year)	Study/Population	Total No. (No. Cases)	Follow-up (Years)	Association 1
Dong *et al.* (2011) [3]	Meta-analysis of 13 studies through 2011	536,318 (24,516)	4–20	RR = 0.78 (0.73–0.84)
Schulze *et al.* (2007) [49]	Meta-analysis of 8 studies through 2006	271,869 (9192)	4–16	RR = 0.77 (0.72–0.84)
Larsson and Wolk (2007) [44]	Meta-analysis of 7 studies 1966–2007	286,668 (10,915)	4–17	RR per 100 mg/day = 0.85 (0.79–0.92)
Hruby *et al.* (2013) [50]	~54 years old; Framingham Heart Study (US)	2582 (179)	7	RR = 0.49 (0.27–0.88), *p* trend = 0.01
Hopping *et al.* (2010) [51]	45–75 years old; Multi-Ethnic Cohort Study (US)	75,512 (8587)	14	Men HR = 0.77 (0.70–0.85), *p* trend < 0.0001; Women HR = 0.84 (0.76–0.93), *p* trend = 0.0003
Kim *et al.* (2010) [42]	18–30 years old; Coronary Artery Risk Development in Young Adults (US)	4497 (330)	20	HR = 0.53 (0.32–0.86), *p* trend < 0.01
Kirii *et al.* (2010) [52]	40–65 years old; Japan Collaborative Cohort Study for Evaluation of Cancer Risk (Japan)	17,592 (459)	5	OR = 0.64 (0.44 to 0.94), *p* trend = 0.04
Nanri *et al.* (2010) [53]	45–75 years old; Japan Public Health Center-based Prospective Study (Japan)	59,791 (1114)	5	Men OR = 0.86 (0.63–1.16), *p* ≥ 0.05; Women OR = 0.92 (0.66–1.28), *p* ≥ 0.05
Villegas *et al.* (2009) [47]	~50 years old; Shanghai Women’s Health Study (China)	64,191 (2270)	7	HR = 0.80 (0.68, 0.93), *p* trend < 0.0001
Schulze *et al.* (2007) [49]	35–65 years old; EPIC–Potsdam (Germany)	25,067 (844)	11	RR = 0.90 (0.72–1.12), *p* trend = 0.44
He *et al.* (2006) [54] ^2^	18–30 years old; Coronary Artery Risk Development in Young Adults (US)	4637 (226)	15	HR = 0.51 (0.32–0.83), *p* trend < 0.01
van Dam *et al.* (2006) [48]	~38 years old; Black Women’s Health Study (US)	41,186 (1964)	8	HR = 0.65 (0.54–0.78), *p* trend < 0.0001
Lopez-Ridaura *et al.* (2004) [45]	~46 years old; Nurses’ Health Study (US)	85,060 (4085)	18	RR = 0.66 (0.60–0.73), *p* trend < 0.001
Lopez-Ridaura *et al.* (2004) [45]	~54 years old; Health Professionals’ Follow-up Study (US)	42,872 (1333)	12	RR = 0.67 (0.56–0.80), *p* trend < 0.001
Hodge *et al.* (2004) [55]	~54 years old; Melbourne Collaborative Cohort Study (Australia)	31,641 (365)	4	OR per 500 mg/day = 0.62 (0.43–0.90)
Song *et al.* (2004) [41]	~54 years old; Women’s Health Study (US)	39,345 (918)	6	RR = 0.89 (0.71–1.10), *p* trend = 0.05
Meyer *et al.* (2000) [56]	~61.5 years old; Iowa Women’s Health Study (US)	35,988 (1141)	6	RR = 0.67 (0.55–0.82), *p* trend = 0.0003
Kao *et al.* (1999) [57]	~53 years old; Atherosclerosis Risk in Communities (US)	12,128 (1106)	6	Black OR = 1.02 (0.58–1.76) ^3^, *p* trend = 0.68; White OR = 0.93 (0.67–1.29) ^3^, *p* trend = 0.84

^1^ Reporting the multivariate-adjusted association (95% confidence interval) for high *versus* low intake, unless otherwise specified; ^2^ Primary study outcome was metabolic syndrome, of which impaired fasting glucose and/or type 2 diabetes was included as a component; ^3^ Highest intake category is reference category; presenting association reported for lowest intake category. HR, hazard ratio; OR, odds ratio; RR, relative risk.

**Table 2:** Cross-sectional studies of magnesium intake and type 2 diabetes or glycemia-related traits.

Author (Year)	Study/Population	No.	Outcome and Association ^1^
Hruby *et al.* (2013) [58]	Meta-analysis of 15 studies (US and Europe)	52,684	FG β per 50 mg/day: −0.009 mmol/L (−0.013, −0.005), *p* < 0.0001; FI β per 50 mg/day: −0.020 ln-pmol/L (−0.024, −0.017), *p* < 0.0001
Cahill *et al.* (2013) [59]	~43 years old; Complex Diseases in the Newfoundland Population: Environment and Genetics Study (Canada)	2295	FG low *vs.* high intake: 5.18 *vs.* 5.17 mmol/L, *p* trend ≥ 0.05; FI low *vs.* high intake: 72.8 *vs.* 60.6 pmol/L, *p* trend < 0.001; HOMA-IR low *vs.* high intake: 2.5 *vs.* 2.1 units, *p* trend = 0.003; HOMA-β low *vs.* high intake: 142.4 *vs.* 116.2 units, *p* trend < 0.001
McKeown *et al.* (2008) [60] ^2^	~72 years old (elderly) (US)	535	IFG/T2D OR high *vs.* low intake: 0.41 (0.22–0.77), *p* trend = 0.005
Ford *et al.* (2007) [61] ^2^	~43 years old; National Health and Nutrition Examination Survey (US)	7669	IFG/T2D OR high *vs.* low intake: 0.85 (0.57–1.28), *p* trend = 0.371
Bo *et al.* (2006) [62]	45–64 years old; (Italy)	1653	T2D OR low *vs.* high intake: 4.3, *p* trend < 0.001; HOMA-IR low *vs.* high intake: 0.5 *vs.* 0.4 units, *p* trend < 0.001; FG ^3^ low *vs.* high intake: 112.1 *vs.* 99.8 mg/dL, *p* trend < 0.001; FI ^3^ low *vs.* high intake: 1.9 *vs.* 1.7 uU/mL, *p* trend < 0.001
Rumawas *et al.* (2006) [35]	~54 years old; Framingham Heart Study (US)	2708	FG low *vs.* high intake: 94.8 *vs.* 94.9 mg/dL, *p* trend = 0.41; FI low *vs.* high intake: 29.9 *vs.* 26.7 uU/mL, *p* trend < 0.001; 2h OGTT glucose low *vs.* high intake: 104.4 *vs.* 100.7 mg/dL, *p* trend = 0.04; 2h OGTT insulin low *vs.* high intake: 86.4 *vs.* 72 mU/mL, *p* trend < 0.001; HOMA-IR low *vs.* high intake: 7.0 *vs.* 6.2 units, *p* trend < 0.001
Song *et al.* (2005) [63] ^2^	~52 years old; Women’s Health Study (US)	9887	Prevalence ^3^ IFG/T2D low *vs.* high intake: 5.0% * vs.* 3.3%, *p* trend = 0.005
Huerta *et al.* (2005) [38] ^4^	~13 years old (US)	48	Correlation, *r*, HOMA-IR: −0.43 (−0.64 to −0.16), *p* = 0.002; Correlation, *r*, FI: −0.43 (−0.64 to −0.16), *p* = 0.002; Correlation, *r*, QUICKI: 0.43 (0.16-0.64), *p* = 0.002; Correlation, *r*, IS: Not significant, association not specified
Song *et al.* (2004) [41]	~55 years old; Women’s Health Study (US)	349	Geometric mean FI low *vs.* high intake: 42.1 *vs.* 38.5 pmol/L, *p* trend = 0.08; BMI ≥ 25 kg/m^2^: 53.5 *vs.* 41.5 pmol/L, *p* trend = 0.03; BMI < 25 kg/m^2^: 34.8 *vs.* 33.0 pmol/L, *p* trend = 0.22
Fung *et al.* (2003) [36]	45–60 years old; Nurses’ Health Study (US)	219	Geometric mean FI low *vs.* high intake: 11.0 *vs.* 9.3 μU/mL, *p* trend = 0.04
Ma *et al.* (1995) [43]	45–64 years old; Atherosclerosis Risk in Communities (US)	15,248	Mean difference FI high *vs.* low intake: White men, 13 pmol/L, *p* < 0.001; Black men, 2 pmol/L, *p* = 0.72; White women, 12 pmol/L, *p* < 0.001; Black women, 27 pmol/L, *p* < 0.001; Mean difference FG high *vs.* low intake: Not specified
Manolio *et al.* (1991) [64]	18–30 years old; Coronary Artery Risk Development in Young Adults (US)	3287	Correlation, *r*, FI: −0.08 to −0.13, *p* < 0.01; FI β per mg/1000 kcal: −0.0006 ln-μU/mL, *p* = 0.0006

^1^ In a given line, the outcome is listed first, followed by the multivariate-adjusted association [e.g., beta coefficient (β), odds ratio (OR), *etc.*], as specified. FG, fasting glucose; FI, fasting insulin; HOMA-β or -IR, homeostasis model assessment of β-cell function or insulin resistance; IFG, impaired fasting glucose; IS, insulin sensitivity; OGTT, oral glucose tolerance test; OR, odds ratio; QUICKI, quantitative insulin sensitivity check index; T2D, type 2 diabetes. ^2^ Primary study outcome was metabolic syndrome, of which impaired fasting glucose and/or type 2 diabetes was included as a component. ^3^ Unadjusted or crude association. ^4^ Case-control study.

Prospective studies [41,42,45,47,48] have observed that individuals with high magnesium intake are 10%–47% less likely to develop type 2 diabetes. A 2011 meta-analysis of 13 prospective cohort studies concluded that the relative risk of type 2 diabetes was 0.78 (95% CI 0.73–0.84) [dose response analysis: per 100 mg/day increment of magnesium intake, the relative risk was 0.86 (95% CI 0.82–0.89)], and authors suggested that the inverse association was stronger in overweight/obese than normal-weight individuals [3]. However, weight status alone may not be a sufficient differentiator in explaining magnesium’s effects on type 2 diabetes risk: two recently published studies investigating serum magnesium (albeit not dietary magnesium), observed that serum magnesium appears to be related to glycemic control independent of obesity. One of these studies was a cross-sectional investigation of serum magnesium in metabolically healthy obese (*vs.* metabolically unhealthy obese) and metabolically obese normal weight (*vs.* metabolically healthy normal weight) individuals. The authors observed that metabolic health [e.g., absence of hypertension, hypertriglyceridemia, hyperglycemia, or insulin resistance (IR)], rather than obesity, *per se*, was associated with hypomagnesaemia [65]. Evidence from another study supporting a favorable link between magnesium and metabolic health investigated the effect of bariatric surgery in obese individuals with and without diabetes on serum magnesium concentrations. The authors observed that after surgery, serum magnesium increased only in individuals in whom diabetes was resolved, but remained unchanged in those for whom diabetes remained, irrespective of differences in weight loss [66].

Few studies have prospectively evaluated magnesium intake and insulin sensitivity or resistance over the long term (*i.e.*, >5 years). One prospective investigation of magnesium intake in 1036 US adults (56.4% women) participating in the Insulin Resistance Atherosclerosis Study who were free of initial and incident type 2 diabetes, estimated that the optimal magnesium intake in relation to insulin sensitivity was at least 325 mg/day [40]. The authors observed progressively poorer insulin sensitivity below that threshold, but no evidence for improvement of sensitivity above that threshold. In this study, insulin sensitivity was assessed by intravenous glucose tolerance tests, considered to be a criterion test for this measure of insulin metabolism. Another study of young Americans, 18–30 years old at baseline and followed for 20 years, observed lower averaged HOMA-β or IR (homeostasis model assessment of β-cell function or insulin resistance) over this time period in those with higher magnesium intake [42]. In our own investigation in the Framingham Heart Study Offspring, we observed a 6.9% incident of diabetes over 7 years of follow-up. Those with the highest magnesium intake had 51% lower risk of developing diabetes than those with the lowest intake, and there was a significant trend across increasing quartile categories of intake in these middle-aged adults, even after adjustment for a number of risk and lifestyle factors, including dietary fiber intake. We also examined HOMA-IR in those 2185 subjects who did not develop diabetes, and observed significant inverse trends with higher magnesium intake and subsequent HOMA-IR in risk factor-adjusted models. However, the trend in these seemingly metabolically healthy individuals was attenuated after additionally adjusting for dietary fiber [50].

A small body of clinical evidence supports a role for magnesium supplementation in glucose and insulin metabolism (Table 3). A meta-analysis of nine magnesium supplement trials in those with type 2 diabetes found that a median magnesium dose of 360 mg/day was associated with significantly lower post-intervention fasting glucose in the treatment groups, suggesting improved glucose control [2]. A recent randomized, placebo-controlled trial in 25 obese, non-diabetic, normo-magnesaemic individuals who supplemented with 365 mg/day of magnesium for six months improved plasma fasting glucose from 5.07 to 4.75 mmol/L, fasting serum insulin from 109.42 to 100.00 pmol/L, the Matsuda insulin sensitivity index from 3.43 to 4.04, and HOMA-IR from 3.49 to 2.97, differences that were not evident in the 22 placebo controls, in whom no improvements were observed [33]. Interestingly, Gutt’s insulin sensitivity index, 2-h post-OGTT glucose, and 2-h post-OGTT insulin did not change significantly between groups [33] suggesting that glycemic homeostasis was affected by the intervention, but not glycemic response. Another recent cross-over supplementation trial in 14 overweight but otherwise healthy adults also showed that 4 weeks of 500 mg/day of magnesium citrate led to lower *C*-peptide and fasting insulin concentrations [12], suggesting reduced pancreatic insulin secretion which could have resulted from improved insulin sensitivity and subsequent lowered demand on the pancreas. Supplementation with magnesium in individuals with other risk factors, such as mild hypertension or hypomagnesaemia, has also been found to be effective in improving insulin sensitivity and pancreatic β-cell function [31,32,37]. In these studies, each of which lasted three months, supplemental magnesium doses ranged from 300 to 600 mg/day. Finally, in a small sample of six healthy, normomagnesaemic participants insulin sensitivity was mildly, but significantly reduced in all subjects (as measured by modified intravenous glucose tolerance test) after three weeks on a low-magnesium diet (3.69 ± 0.6 *vs.* 2.75 ± 0.5 min^−1^ per μU/mL × 10^−4^, *p* < 0.03, paired analysis). Interestingly, neither fasting glucose nor fasting insulin concentrations were significantly changed by this low magnesium diet [67].

## 5. The Genetics of Magnesium Homeostasis

Despite magnesium’s tight homeostatic regulation, surprisingly little is known about the mechanisms that are broadly regulating total body magnesium in humans, perhaps owing to the complexity and ubiquity of magnesium and its roles in the body. Nevertheless, among the most investigated of the known genes implicated in magnesium transport and homeostasis are the variants in transient receptor potential cation channel, subfamily M (*TRPM*), members 6 (*TRPM6*) or 7 (*TRPM7*), which encode magnesium transporters: *TRPM6* expression occurring mainly in the kidney and intestine, where it is critical for reabsorption, while *TRPM7* expression occurs ubiquitously [68]. After the *TRPM*s, additional mutations involved in familial hypomagnesemia (with or without hypocalcemia or hypercalciuria), such as mutations in *CLDN16* and *CLDN19* (which encode tight junction proteins claudin-16 and claudin-19 and regulate aspects of ion reabsorption in the kidney) [69], mutations in the solute carrier families 12 (*SLC12A3*—encoding a sodium/choride transporter) and 41 (*SLC41A1* and *SLC41A2*—which mediate magnesium transport across membranes; interestingly the former is the only magnesium-responsive gene overexpressed in placental tissue of preeclamptic women [70]), magnesium transporter protein 1 (*MagT1*—another magnesium transporter; implicated in immune activation), among others. Functional and experimental studies on a number of these genes are extensive, and have been expertly reviewed by several authors [71,72,73].

**Table 3:** Trials of magnesium and type 2 diabetes and related outcomes.

Author (Year)	Design/Population ^1^	No., Tx (control)	Mg Tx *vs.* Control	Post-Tx Effects
Song *et al.* (2006) [2]	Meta-analysis of 9 RCTs through January 2005, 4–16 week durations	370 with T2D	Median 360 mg/day	FG: decreased; HbA1c: no change
Guerrero-Romero and Rodriguez-Moran (2011) [37]	12 week; hypomagnesaemic, overweight; ~40 years old (Mexico)	49 (48)	2.5 g/day MgCl_2_ (solution); 50 mL inactive solution	FG: decreased; FI: decreased; Belfiore index: improved; HOMA-β: decreased only in placebo; serum Mg: increased
Hadjistavri *et al.* (2010) [32]	12 week; mild hypertensive, overweight; ~45 years old (Greece)	24 (24)	600 mg/day Mg pidolate (solution); lifestyle recommendations	FG: no change; FI: decreased; HOMA-IR: decreased; Cedercholm index: increased; Matsuda index: increased; Stumvoll index: increased; AUC glucose: decreased; AUC insulin: decreased; serum and 24h urine Mg: increased
Lee *et al.* (2009) [74]	12 week; healthy, normo-magnesaemic, overweight; 30–60 years old (Korea)	75 (80)	12.2 mmol (300 mg) as MgO; placebo	FG: no difference between groups; FI: no difference between groups; HOMA-IR: no difference between groups; serum Mg: no change (except in those with hypertension)
Guerrero-Romero *et al.* (2004) [31]	12 week; hypomagnesaemic, insulin resistant, overweight; ~42.5 years old (Mexico)	30 (30)	2.5 g/day MgCl_2_ (solution); 50 mL inactive solution	FG: decreased; FI: decreased; HOMA-IR: decreased; serum Mg: increased
Rodriguez-Moran and Guerrero-Romero (2003) [75]	16 week; T2D, hypomagnesaemic; ~56 years old (Mexico)	32 (31)	50 g MgCl_2_ (50 mL solution); placebo	FG: decreased; FI: increased; HbA1c: decreased; HOMA-IR: decreased; serum Mg: increased
Chacko *et al.* (2010) [12]	Randomized, double-blind, crossover; 4 week; 4 week washout; healthy, overweight; ~44.4 years old (US)	13	500 mg/day elemental Mg as Mg citrate; placebo	FG: No change; HbA1c: increased (*p* = 0.08); FI: no change; *C*-peptide: decreased; serum Mg: no change
Paolisso *et al.* (1994) [76]	Randomized, double-blind, crossover; 4 week; 3 week run-in; T2D, elderly (Italy)	9	15.8 mmol/day; placebo	FG: No change; glucose disposal: increased; glucose oxidation: increased; plasma and erythrocyte Mg: increased
Purvis *et al.* (1994) [77]	Randomized, double-blind, crossover; 6 week; 2 week run-in, 2 week washout; T2D, hypercholesterolemic; ~53.8 years old (US)	28	384 mg/day MgCl (Slo-Mag); placebo	FG: no change; serum Mg: no change
Paolisso *et al.* (1992) [34]	Randomized, double-blind, crossover; 4 week; 4 week run-in, 2 week washout; generally healthy, non-obese; ~77.8 years old (Italy)	12	4.5 g/day Mg pidolate (16.2 mmol Mg); placebo	FG: decreased; FI: no change; acute and total insulin response: increased; glucose disappearance: improved; hepatic glucose output: no difference; glucose uptake: improved; plasma and erythrocyte Mg: increased
Paolisso *et al.* (1989) [78]	Randomized, crossover; 4 week; 3 week run-in, 2 week washout; T2D, moderately obese; ~67 years old (Italy)	8	3 g/day as Mag 2	FG: decreased; acute and total insulin response: increased; glucose disappearance: improved; plasma and erythrocyte Mg: improved
Paolisso *et al.* (1989) [79]	Randomized, crossover; 4 week; 3 week run-in, 2 week washout; T2D, moderately obese; ~67 years old (Italy)	8	2 g/day as Mag 2; placebo	FG: decreased; acute insulin response: increased glucose infusion rate: increased; plasma and erythrocyte Mg: improved
Yokota *et al.* (2004) [46]	Uncontrolled supplementation study; 30 day; mild T2D (no insulin); ~51.6 years old (Japan)	9	300 mg/day as mineral water (Mag21, “bittern”); n/a	FG: No change; HbA1c: no change; FI: 8.08 to 5.89 μU/mL, *p* ≤ 0.05; HOMA-IR: 2.73 to 2.05 units *p* ≤ 0.05; serum and urinary Mg excretion: increased
Nielsen *et al* (2007) [10]	Depletion/repletion; depletion: ≤78 day; repletion: ≥58 day; healthy, post-menopausal; 47–75 years old (US)	14	Depletion: 33% of Mg RDA (101 mg/2000 kcal/day) diet; repletion: diet plus extra 200 mg/day	AUC glucose: higher during depletion than repletion; AUC insulin: no change; erythrocyte Mg: initial increase, then decrease during depletion; serum Mg: initial decrease, then increase during depletion
Nadler *et al.* (1993) [67]	Depletion; 3 week; presumably healthy, overweight (US)	12	Liquid diet: 1 week with 400 mg/day MgCl_2_ followed by 3 week with low Mg [12 mg/day (<0.05 mmol/day)]; n/a	FG: no change; FI: no change; Bergman index: decreased; serum and intracellular Mg: decreased

^1^ Randomized, controlled trial, unless otherwise specified; AUC, area under the curve; FG, fasting glucose; FI, fasting insulin; HOMA-β or -IR, homeostasis model assessment of β-cell function or insulin resistance; IS, insulin sensitivity; Mg, magnesium; OGTT, oral glucose tolerance test; QUICKI, quantitative insulin sensitivity check index; RDA, recommended dietary allowance; T2D, type 2 diabetes; Tx, treatment. n/a, not applicable (no placebo).

Two additional studies in humans, both published in 2010, have spurred ongoing interest in the genetics of magnesium. One of these was a meta-analysis of genome-wide association studies (GWAS) of serum magnesium (as well as serum potassium and sodium), originally designed to evaluate the contribution of common genetic variation to the normal physiologic variation in serum concentrations [80]. No significant hits were found for sodium or potassium. However, six regions, while collectively only contributing to 1.6% of the variability in serum magnesium concentrations, were identified as top hits for magnesium (in or near *MUC1*, *ATP2B1*, *DCDC5*, *TRPM6*, *SHROOM3*, and *MDS1*). Nevertheless, because the top six polymorphisms seem to involve magnesium transport and homeostasis, it is plausible to hypothesize that they may also modify individual risk for magnesium-related conditions—including diabetes—in the face of differential magnesium intake.

Finally, gene-expression profiles following four weeks of magnesium supplementation in a pilot trial with cross-over design in 14 overweight but otherwise healthy individuals, revealed that supplementation up-regulated 22 genes, and down-regulated 36 by 20% or more as compared to placebo, many of these involved in inflammatory pathways [12]. Most interestingly, perhaps, over half of the differentially regulated regions were of unknown function. The potential epigenetic effects of magnesium deficiency or supplementation in inflammation are supported in part by studies in rats of methylation of promoter regions of 11β-hydroxysteroid dehydrogenase-2 (*Hsd11b2*). In the offspring of magnesium-deficient dams, hepatic *Hsd11b2* CpG promoters showed substantial hyper-methylation, likely pointing to downstream down-regulated gene expression [81]. Note that *HSD11B1* gene deficiency (whose promoter region is hypo-methylated in calcium-deficiency in rats [82]) and *HSD11B2* gene overexpression are associated with improvements in metabolic characteristics such as those related to hypertension and diabetes.

While many genetic interaction studies, such as the ones we discuss in greater detail below, are “nutrigenetic” in that they reveal how or whether polymorphisms modify how we use nutrients, “nutrigenomics” studies such as the cross-over trial above reveal broader genetic response to magnesium supplementation as an environmental stimulus, revealing the complexity of our responses to environmental exposures. Hence the latter are hypothesis-generating, while results from the former may perhaps be more amenable to dictating personalized approaches to health and disease management.

### Interactions of Dietary Magnesium and Genes in Glucose and Insulin Homeostasis and Metabolism

In humans, only four studies have thus far specifically investigated associations between loci in many of the candidate genes discussed above and diabetes or glycemic traits [58,68,83,84]. In 2009, Song *et al.* [84], first examined 20 haplotype-tagging single nucleotide polymorphisms (SNPs) in *TRPM6* and 5 common SNPs in *TRPM7* for their association with diabetes risk. They reported that two common non-synonymous *TRPM6* coding region variants—Val1393Ile in exon 29 (rs3750425) and Lys1584Glu in exon 30 (rs2274924)—might confer susceptibility to type 2 diabetes in women with low magnesium intake. Women who were carriers of the two rare alleles of rs3750425 and rs2274924 had nearly five times the odds of type 2 diabetes compared to women who were non-carriers, only when their magnesium intake was <250 mg/day. Two amino acid changes by these polymorphisms are located between the coiled region and kinase near the *C*-terminal and may reduce *TRPM6* channel activity by changing protein conformation. However, the other three studies to date which have investigated such relationships have found that magnesium-related variants did not individually modify either risk of type 2 diabetes [68,84] or glycemic traits [58,68]. Two of these studies [58,84] have also examined interactions of dietary magnesium intake with select risk loci implicated in diabetes- or magnesium-related traits. Again, neither study found strong evidence for interaction with any individual locus and magnesium intake. Recently, Nair *et al.* [83] conducted an association study to examine the relation between these two functional polymorphisms and gestational diabetes. They observed a significant association between carriers of the *TRPM6* rs2274924 variant and elevated total glycosylated hemoglobin and greater prevalence of gestational diabetes in 997 women following delivery. They also conducted functional testing to explore the functionality of this SNP. The authors reported that the insulin signaling cascade is unable to activate the phosphorylation of the amino acid adjacent to the substituted amino acid resulting from the polymorphism, thereby rendering the variant TRPM6 channel insensitive to the activating effects of insulin.

Hruby *et al.* [58] reported a dietary magnesium-gene interaction study involving over 50,000 individuals free of diabetes from 15 cohorts in the US and Europe, which was the largest of the dietary magnesium-gene interaction studies conducted to date. Dietary intake in these cohorts was generally measured by food frequency questionnaire, and the loci selected for interaction analyses had been previously identified in GWAS as being related to either serum magnesium, or fasting glucose or insulin in non-diabetic individuals. In this study, no magnesium-related locus (including loci in *TRMP6*, *TRPM7*, *CNNM2* and *MUC1*) was significantly associated with either fasting glucose or fasting insulin, and none of the eight magnesium-locus × dietary-magnesium interactions were statistically significant. However, the authors observed suggestive inverse association of rs2274924 and rs3750425 in *TRPM6* with fasting glucose, the same loci associated with risk of diabetes in women when in haplotype [84], and with risk of gestational diabetes [83]. The non-significant findings which nevertheless show some consistency with concurrent and prior work in this area, lend further plausibility to a role for *TRPM6* in glycemia and diabetes. It is possible that in these studies, all of which have been observational, there is some unknown or uncontrolled for confounder which is obscuring a more clear relationship between the loci and the glycemia-related outcomes.

In this meta-analysis, one suggestive interaction between magnesium intake and fasting glucose, was observed with rs3740393 in *CNNM2*. *CNNM2* encodes a membrane protein required for renal magnesium handling and the G allele at this locus was associated with lower serum magnesium [80]. If replicated in future studies, the interaction suggests that the magnitude of the inverse association between magnesium intake and fasting glucose is diminished in the presence of the serum magnesium-lowering G allele, *versus* the C allele. This interaction potentially indicates a higher dietary magnesium requirement in those with a propensity for lower serum magnesium, for beneficial effects on fasting glucose.

## 6. Conclusions

Type 2 diabetes is a complex, multifactorial disorder, heavily influenced by both genetic and environmental determinants. Yet neither genetics nor environmental factors alone explain why some develop the disease while others don’t, thus paving the way for hypotheses on environment-gene interactions, most notably interactions involving modifiable lifestyle factors such as physical activity and diet.

Findings from dietary magnesium-gene investigations conducted to date have generally failed to yield consistent and robust results. Interaction analyses have suffered from a number of limitations that may hamper the accumulation of unequivocal evidence. As has been frequently noted by those working in the field of nutrigenetics/nutrigenomics [85] at the epidemiologic level, such work is inherently complex, likely involving multiple loci, biomarkers, and biofeedback, and yet thus far statistical modeling has taken a relatively simple approach (e.g., a single locus, haplotype, or genetic risk score based on multiple loci, crossed with a single measure of the environmental or dietary exposure). Studies are prone to spurious associations, and signals are potentially overwhelmed by residual confounding, imprecise dietary measures, or multiple testing.

Future well-designed mechanistic studies, which would lend themselves to our basic understanding of magnesium homeostasis, are warranted. Among these is a relatively straightforward study of magnesium supplementation in carriers of rare variants associated with magnesium homeostasis and transport, notably the *TRPM*s as well as familial hypomagnesaemia, and how supplementation in these individuals differentially affects biomarkers of magnesium status relative to those without the mutations. Such a study would do much to not only assess the magnitude of the impact of serum magnesium-lowering alleles in the presence of equivalent dietary intake in non-carriers, but also the underlying mechanisms.

A second and equally obvious trial is that of magnesium supplementation as primary prevention for diabetes. To date, such a study has been unfeasible owing to length-of-follow-up and costs associated with long-term randomized trials. Hampering this and other studies of single supplemental nutrients is the historicity of the single-nutrient approach in the context of the randomized trial: many have notoriously not wrought the fruit suggested they would by observational literature. Nevertheless, other observational approaches designed to advance our understanding of magnesium-gene interactions are underway, including a genome-wide interaction study of magnesium intake involving over 20 observational cohorts in the US and Europe. In the interim, then, these and functional and mechanistic studies, coupled with smaller trials in genetically or physiologically at-risk individuals will continue to be the norm, and will hopefully lend themselves to the incremental insights needed to truly understand the myriad roles of this fascinating and yet relatively underappreciated mineral.
